# Using machine learning to predict and analyze complex trait diseases: Lessons from a simple abstract model

**DOI:** 10.1371/journal.pone.0342490

**Published:** 2026-02-23

**Authors:** Eden Maimon, Ori Bondi, John Moult, Ron Unger

**Affiliations:** 1 The Mina and Everard Goodman Faculty of Life Sciences, Bar-Ilan University, Ramat-Gan, Israel; 2 Institute for Bioscience and Biotechnology Research, Rockville, Maryland, United States of America; 3 Department of Cell Biology and Molecular Genetics, University of Maryland, College Park, Maryland, United States of America; Nottingham Trent University School of Science and Technology, UNITED KINGDOM OF GREAT BRITAIN AND NORTHERN IRELAND

## Abstract

The ability to predict individual genetic susceptibility to a complex trait disease is a major challenge in modern medicine. One approach to addressing this challenge utilizes an additive combination of contributions from a large number of single nucleotide polymorphisms (SNPs), with weights derived from Genome Wide Association Studies (GWAS). While this approach is somewhat successful in predicting *whether* an individual is likely to develop a specific disease, it does not explain *why* a person is likely to become sick. Here, we designed and utilized abstract disease models to investigate the relationship between disease structure, susceptibility, and predictability. The model consists of a set of interacting pathways, each including several nodes representing loci at which genetic variants can alter the function of the corresponding proteins. Due to the introduction of thresholds for pathway functionality, and the interplay between the pathways, this model is inherently non-additive. We use this “toy model” together with simulated variant data to examine the effect of changing various properties, some of which cannot be easily controlled in a “real-world” scenario. As expected, larger sample sizes improved the performance; the omission of some contributing variants from the dataset was associated with a significant decrease in performance, whereas adding irrelevant variants had little effect. Surprisingly, diseases with a more complex underlying structure were better predicted than those with a simpler structure. In addition, risk prediction was more accurate for diseases with lower prevalence. The algorithm was robust to a reasonable percentage of false negative disease assignments. The largest decrease in performance occurred when two diseases with different genetic etiologies were classified as a single pathology, as often occurs in clinical situations, and apparently confuses the neural network algorithm. Finally, we show that a post-analysis of a neural network using t-SNE can provide biological insights into the underlying disease structure.

## Introduction

Understanding the relationship between genotype and phenotype is a holy grail of modern biology. In recent years, there has been a dramatic improvement in our ability to determine variants in individual human genomes, first by using microarray chip technology and subsequently by partial or full genome sequencing. These technologies have greatly improved understanding of genotype/phenotype relationships for a broad range of Mendelian diseases [1], cancer [2], and complex trait diseases [3]. The latter class includes many of the most common human diseases such as Cardiovascular disease (CVD),

Alzheimer’s Disease, Parkinson’s Disease, and Type 2 Diabetes and also poses the greatest interpretation challenge, both because of the interactions of multiple variants and the substantial contributions from environmental and lifestyle factors [4].

For these diseases, Genome Wide Association Studies (GWAS) have discovered many (typically tens to hundreds) of genomic loci statistically associated with specific disease phenotypes. Applications of these data include deriving improved insight into the underlying disease mechanism [5], exploring new therapeutic possibilities [6], and developing models for predicting individual risk of specific diseases [7], or risk of progression to more severe disease [8]. The models also have the potential to facilitate early diagnosis and treatment, improved prognosis, and possible prevention of the disease by lifestyle modification [9,10].

A well-established approach to utilize GWAS data for disease risk prediction is to compute a Polygenic Risk Score (PRS), a weighted sum of contributions from a large number of SNPs, with each SNP representing a disease-associated locus and with weights derived from estimated effect sizes [11]. Each included SNP may represent a damaging or protective effect on the phenotype, and thus, its contribution may marginally increase or decrease the total score. We stress that the terms “damaging” and “protective” should be considered on a statistical level and do not imply causality. Thousands or even millions of SNPS may be included in a PRS score [12,13] and the choice of which and how many to include is a subject of heated debate. The main characteristics of this class of model is that a large number of SNPs contribute to disease risk, and that a simple weighted summation of their contributions provides a useful risk score. The number of significant loci associations discovered has been increasing steadily as study sizes become larger [14], supporting the view that many small contributions may be involved. While PRS models are successful in providing probabilities of an individual displaying a trait based on additive contributions, they do not incorporate any non-additive effects between genetic loci.

Here, we introduce a simple pathway-based model representing underlying disease structure, and explore the ability of machine learning algorithms to predict disease status and structure under a range of scenarios. An important aspect of the model is that it includes non-additive effects arising from pathway properties and interactions.

Recent studies have examined the possibility of utilizing “reverse-engineering’ to determine the disease structure from the predictive models (see for example [15–18]). These models dealt with actual diseases and with real genetic data. Thus, because of limited knowledge of the true underlying disease structure there are no good benchmarks to assess such approaches.

Several “real world” studies were aimed to found out if standard machine learning methods perform better than classical statistical genetics methods [19–21] and whether deep learning algorithms perform better than “classic” machine learning algorithms [20–23] with mixed results, highlighting again the methodological difficulties in using real-life data.

In introducing these simple models, we don’t aim to replace the established quantitative genetic frameworks, but rather to explore whether simple abstract models can capture structural properties of disease that affect machine learning (ML) performance. Neither was our objective to develop a sophisticated competitive prediction method.

Rather, we aimed to investigate the correlation between disease model properties and predictive capacity within a simple abstract clean model. Thus, we sought to develop a “toy-model” that facilitates comprehension of the underlying relationships between the disease structure and the ability to predict an individual’s risk for that disease. This approach is analogous to the successful utilization of a simple lattice model to understand the principles of protein folding (for example [24,25]).

In our model, individuals are represented by their “genomes”, a small number (a few dozens) of loci, wherein for each locus, an individual can be either wildtype, or heterozygous or homozygous for the disease-associated allele. The model is based on representing a disease by a set of pathways, each including several nodes representing loci in which a variant can alter the function of the corresponding proteins. If the accumulation of these variants in a given pathway passes a threshold, the pathway is considered non- functional. When the number of non-functional pathways exceeds a second threshold, then the individual is considered a disease “case”; otherwise he is considered a “control”.

Because of the introduction of thresholds determining the functionality of each pathway, and because of the interplay between the pathways, this model is inherently non-additive. This form of non-additivity is different from the commonly used terms of “negative epistasis”, “positive epistasis” and “reciprocal sign epistasis”. (In the Discussion we address these two forms of non-additivity and show that with certain modifications our model can emulate the more common definition). For a given disease model, we create simulated genotypes of individuals and classify them as “Case” or “Control” according to the model. Then, the genotype data are presented to ML algorithms that are blind to the disease model and are only exposed to the classification status of each individual in the training set. Algorithms are challenged to predict the disease status of individuals in the test set. This setup allows us to determine which factor have the greatest influence on performance. The model overlooks important real-life features such as genetic loci redundancy, family structure, ethnic variation, and potential effects of population stratification. Despite these limitations, the simplicity of this setup makes it possible to study the relationship between disease structure and methods in a way that cannot be done with real data, and to produce hypotheses that could be later tested in real-life scenarios.

We tested several factors related to the underlying disease and its structure, for example, whether it is easier to predict a common or a rare disease, and whether it is more difficult or easier to predict a disease when the underlying structure is more biologically complex. Some of the factors examined are technical and include the size of the data set provided, and the quality of the set of SNPs used (input set features lacking some of the disease- influencing SNPs, or including “false” SNPs that are not associated with disease).

Finally, we investigated whether prediction models can potentially be used to gain mechanistic insights into the underlying disease, reverse-engineering the structure of the disease from the model.

Our model is extremely simplified. Nevertheless, we believe that despite its simplicity, the model can capture the essence of a “real disease” model (see Discussion), and thus provide insight for improved disease prediction.

## Methodology

### The disease model

In our model, an individual’s disease-related **genotype** is mapped to a group of disease related pathways, each composed of several genes that may or may not carry risk- increasing or risk-decreasing SNPs. For simplicity, we assume that each risk-related SNP is associated with a single gene, and vise-versa. A SNP is represented by a numeric value that indicates the type of variants at this locus. Hence, each SNP has the value ‘0’, ‘1’ or ‘2’, representing homozygous-wild type (no variants), heterozygous (only one variant), or a homozygous variant (two variants), respectively. When the SNP is protective (i.e., its frequency in a case population is lower than in the healthy control population), we designate the wild type genotype as the “risk allele”, and the number we consider (0, 1, 2) is the number of risk alleles in the locus. All the subsequent analyses are described in terms of risk alleles, rather than SNPs.

Since complex trait diseases involve numerous genetic loci, all individuals are likely to carry some of the genetic risk alleles for the disease. However, because of the genetic robustness of biological systems [26,27], the majority of the population is not affected, since the combined load of risk alleles does not exceed a risk burden threshold. This robustness protects the system from some genetic perturbations, through various mechanisms such as gene duplication, feedback compensation, alternative pathways, and more long-range network properties, and allows it to function adequately despite the presence of genes with altered activity [27,28].

Given these considerations, the disease model consists of [P] pathways (or modules) with [R] genes in each pathway. The function of each gene could be significantly altered by a single risk allele. For simplicity, we assume that all pathways contain the same number of genes.

An individual is defined as “Case” if the overall number of malfunctioning pathways [**Pe**] exceeds a predefined threshold **M**_**p**_, otherwise, the individual is classified as Control. Similarly, a pathway *i* is defined as “malfunctioning” if the total burden of risk alleles [**Re**_**i**_] in the pathway is greater than a predefined threshold, **M_g_**.

Using these basic definitions, together with additional adjustable parameters, we generated disease models with varying levels of complexity. In each type of model, two major parameters affect complexity. The first is the structure of the genotype; for example, are there risk alleles that participate in several pathways? The second is the magnitude of the risk effect contributed by each malfunctioning pathway and by each risk allele. Accordingly, there may be pathways or risk alleles that have a greater role than others, and thus, perturbation of these has a greater effect. Using these concepts, we established the following models:

### The simple model

In this model, the pathways are independent and there are no risk-alleles that affect more than one pathway. Additionally, all the risk alleles have equal weights, and all the pathways have equal weights.

**Fig 1:**
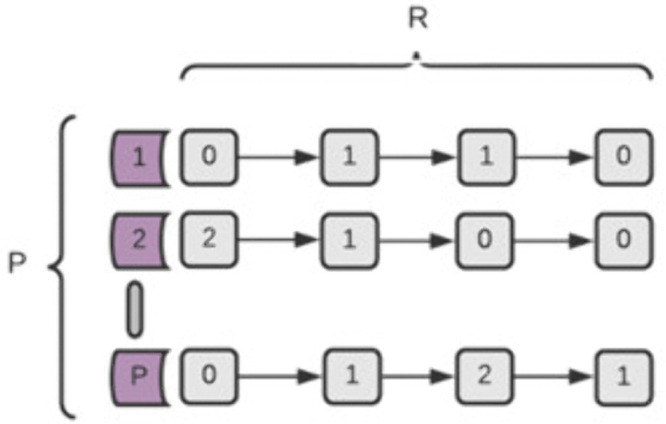
Graphic representation of the Simple disease model of R risk alleles and P pathways.


The contribution of the j’th risk allele to the overall effect on its path is simply its value [**R**_**j**_]. The homozygote risk allele has a contribution of 2, heterozygote risk state contributes 1, and the homozygote protective allele 0. In Fig 1, the total effect of the risk alleles in the i’th pathway [**Re**_**i**_] is the sum of the values of all the risk alleles in that pathway (Eq 1.1). If this value is greater than **M_g_**, then the pathway is defined as “malfunctioning^”^ (Eq 1.2). If the number of malfunctioning pathways (Pe, Eq 1.3) is greater than a threshold Mp, then the individual is considered “Case”, otherwise it is considered “Control” (eq. 1.4) Thus, given a set of risk allele assignments (either 0, 1 or 2) representing an individual, it is possible to assign the values of the alleles into a given disease model and determine if the individual is Case or Control.

$$Re_{i}=\sum_{J}^{R}R_{j}$$ (1.1)

$$Th\left(Re_{i}\right)=\left\{ \begin{array}{cc} @cc@1, & Re_{i}>M_{g}\\ 0, & otherwise \end{array}\right.$$ (1.2)

$$Pe=\sum_{i}^{P}Th({Re}_{i})$$ (1.3)

$$Status\left(Pe\right)=\left\{ \begin{array}{cc} @ll@Case, & Pe>M_{P}\\ Control, & otherwise \end{array}\right.$$ (1.4)

### The weighted model

In this model, not all the genes in a pathway and not all the pathways that participate in the disease are given equal weights. We assume that some pathways are more relevant to the disease than others [29]. Likewise, risk alleles can have varying degrees of influence on their pathways.

To capture these differences in the model, the pathways are divided into two subgroups: “central-pathways” – representing pathways that have a more direct impact on the disease and thus have larger weights, and the remaining pathways, which are considered “peripheral-pathways” with lower weights. Similarly, the risk alleles in each pathway are also divided into “high-risk allele” and “low-risk allele” subgroups with two levels of weights.

**Fig 2:**
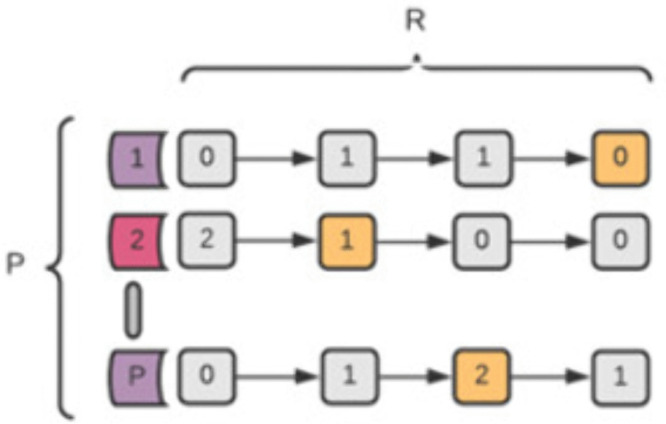
Graphic representation of a Weighted disease model of R risk-alleles and P pathways. The second pathway (red ‘2’) represents a central-pathway and the orange-colored alleles represent the high-risk alleles.


In the Weighted model (Fig 2), the effect of the j^th^ risk allele on its pathway is the product of the risk allele’s value [**R_j_**] and its weight [**R_wj_**] (Eq. 2.1). The same concept is applied to the effect of malfunctioning pathway [**Pe**], where a weight is attached to each pathway [**P_wi_**], and the sum of the weights of all the pathways is compared to the **M_p_** threshold to decide whether the individual is Case or Control (Eq 2.2). **Th** is a threshold used to determine whether a pathway is malfunctioning.

$$Re_{i}=\sum_{J}^{R}Rw_{j}\times R_{j}$$ (2.1)

$$Pe=\sum_{i}^{P}Pw_{i}\times Th\left(Re_{i}\right)$$ (2.2)

### The overlap model

**Fig 3:**
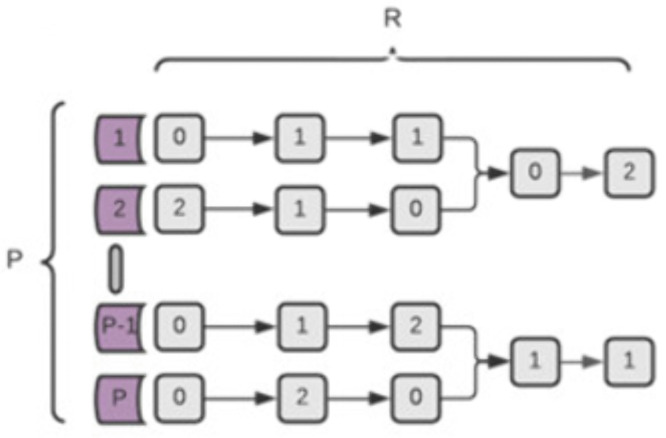
Graphic representation of an Overlap disease model of R risk alleles and P pathways. Pathways are divided into pairs, and each pair shares two SNPs. A shared risk allele value is included in the calculations of both paths.


This model (Fig 3) is a different extension of the Simple model, where all paths and all risk alleles have the same contribution. However, here, the pathways have overlapping risk alleles, i.e., risk alleles may take part in more than one pathway. We assume that these overlapping risk alleles will have a greater influence on disease risk. For simplicity, we built this model such that every path shares two risk alleles with only one other pathway. Re and Pe are calculated in the same way as in the Simple model. **Th** is a threshold used to determine whether a pathway is malfunctioning (Eqs 3.1 and 3.2).

$$Re_{i}=\sum_{J}^{R}R_{j}$$ (3.1)

$$Pe=\sum_{i}^{P}Th(Re_{i})$$ (3.2)

### The subtype model

**Fig 4:**
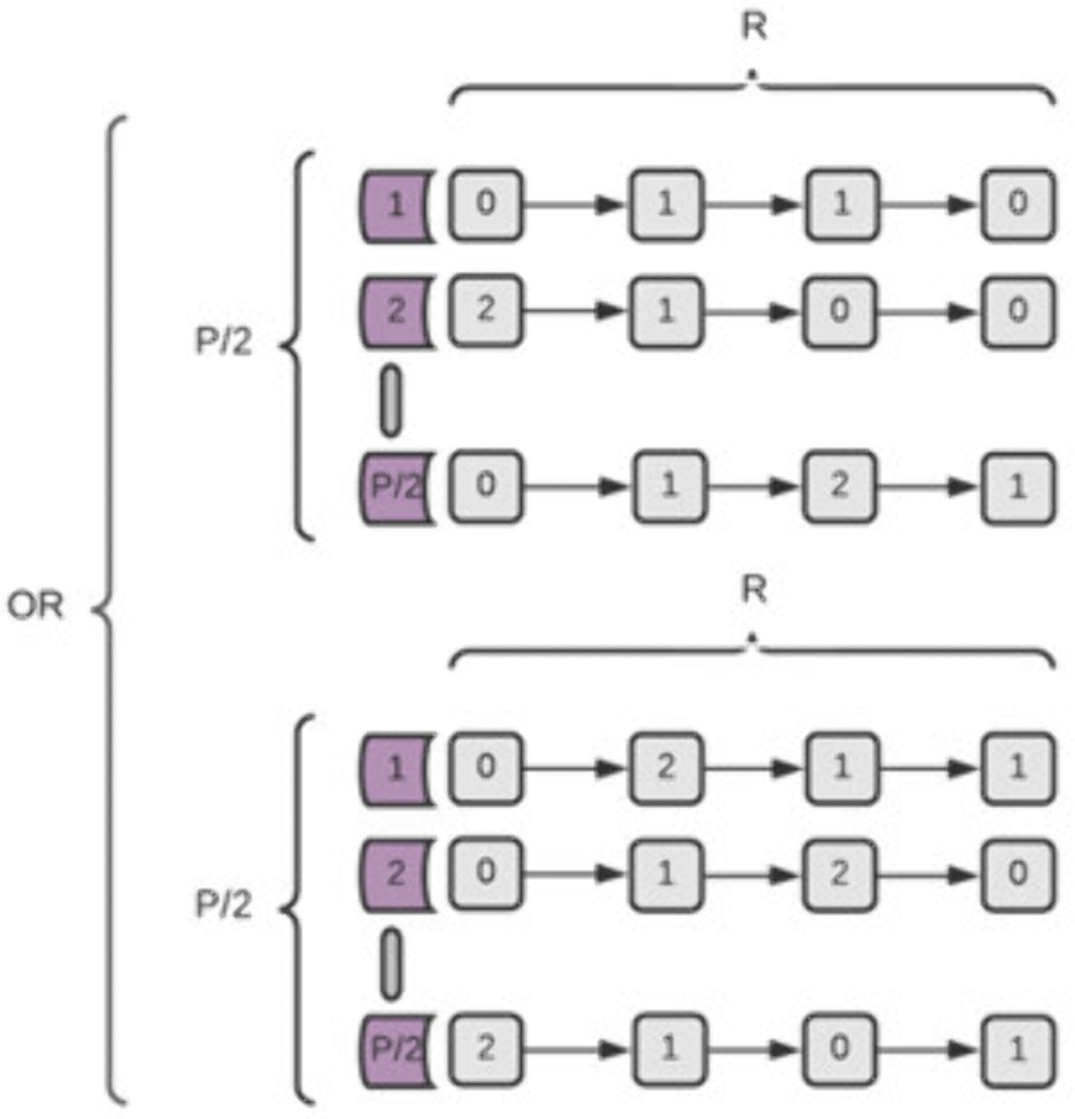
Graphic representation of a Subtype disease model, composed of two alternative Simple models.


This model (Fig 4) simulates a situation in which two genetically different diseases create a similar phenotype and are therefore classified as the same disease [26]. We implemented this scenario by creating two unique, non-overlapping Simple models and using them to classify a set of genotypes (Eq 4.1). An individual is considered Case if classified as such by at least one of the sub-models, otherwise, the individual is defined as Control (Eq 4.2).

$$\begin{array}{c} Pe_{\text{1}}=\sum_{i=1}^{P/2}Th(Re_{i})\mathrm{\backslash vspace}1.5mm\\ Pe_{\text{2}}=\sum_{1+P/2}^{P}Th(Re_{i}) \end{array}$$ (4.1)

$$Status\left(Pe_{\text{1}},Pe_{\text{2}}\right)=\left\{ \begin{array}{c} @l@\;\;Case,\;Pe_{\text{1}}>Mp\text{\textbackslash{}hspace\{0.33em}\}or\text{\textbackslash{}hspace\{0.33em}\}Pe_{\text{2}}>Mp\text{\textbackslash{}hspace\{0.33em}\}\\ Control,\;otherwise \end{array}\right.$$ (4.2)

Assigning numerical values to parameters of the model is somewhat arbitrary but constrained by the need to achieve the appropriate balance of Case and Control individuals. While some deviations from the values used here are possible without qualitatively changing the results, large changes may result in a swing to all Control or all Case individuals.

The supplementary file (S1 File) shows examples of calculating the status of individuals in each model. A glossary of the parameters in the models can be found in Supplementary file S2 File.

### Creating the datasets

Having defined a set of disease models reflecting different complexities of disease structure, we next generated GWAS-like data sets for use in examining the different factors that may affect prediction performance.

To generate the datasets, we started with a core set of data based on a real Crohn’s disease [CD] data-set containing the status of SNPs in 3004 and 1949 Case individuals derived from the WTCCC GWAS study [30]. As mentioned above, the presence of a SNP may be associated with increased or decreased disease risk compared to the wild type. As outlined earlier, we considered “risk” alleles, independently of whether these alleles are actually the SNPs or wild type. For example, if a particular SNP is protective, we consider having the wild type as a “risk” allele. We selected 48 loci from the 163 Crohn’s disease–associated loci in the WTCCC dataset, for which data completeness was highest, 818 missing data values for the sick individuals (0.8%) and 822 points (0.5%) for the healthy individuals. We filled in the few missing values by using genotypes from the most similar samples of the same class. Namely, for each sample with a missing value, we found the closest sample (by Hamming distance) from the same class (Case/ Control) and used their values to complete the missing values in our sample; if the closest sample also had a missing value in the same locus, we used the second most similar sample, and so on. This process resulted in two matrices with one column for each of the 48 risk alleles and one row per individual; one matrix stored the information for Case and one for Control individuals.

**Fig 5:**
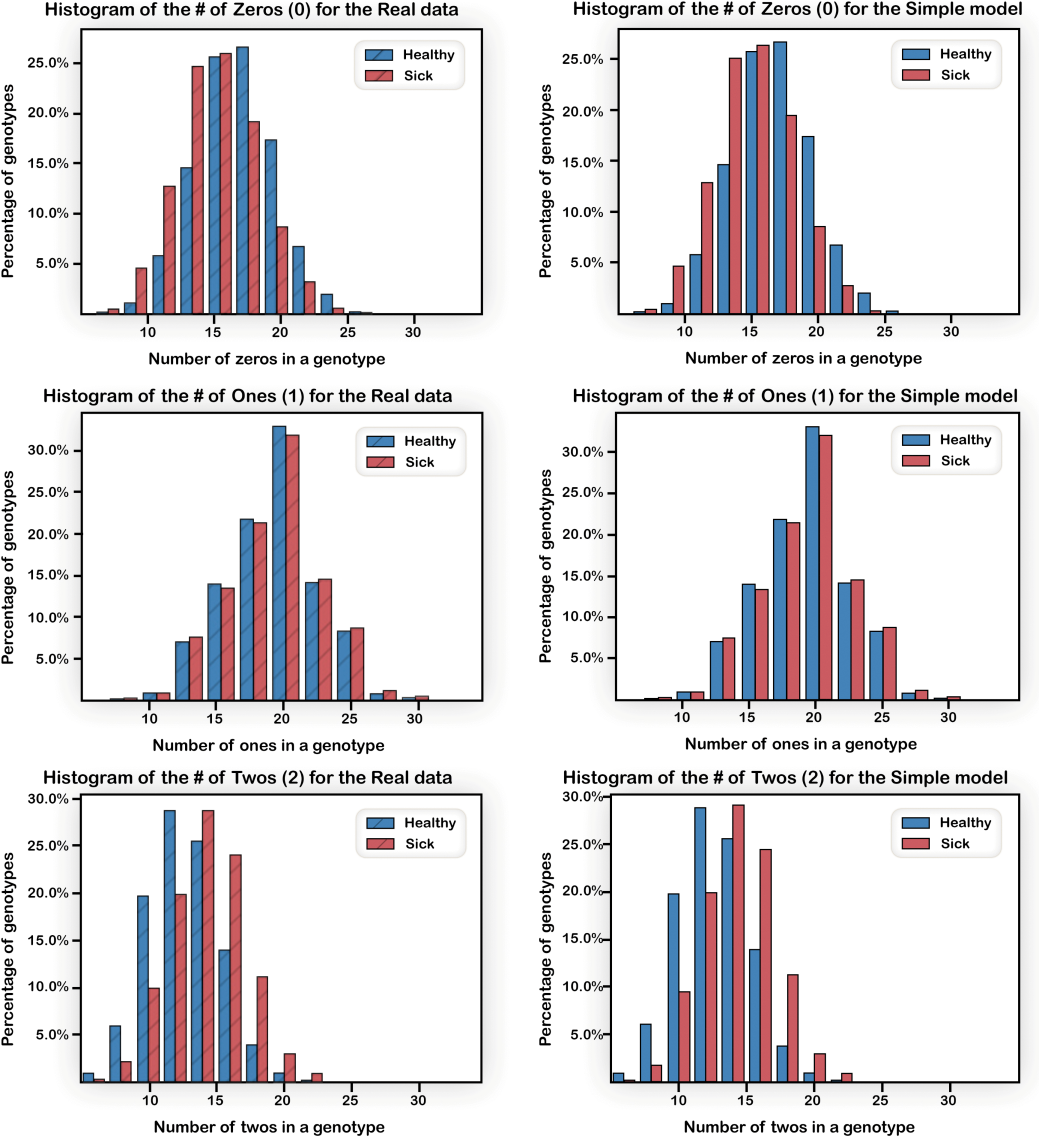
Risk allele histograms for the core Crohn’s disease (CD, 3004 individuals) and Control populations (1949 individuals) in the 48 loci (left), and in a simulated data set (right). The top, middle, and lower panels show the distribution of individuals carrying 0, 1, and 2 risk alleles, respectively. The CD population had a slightly higher fraction of risk alleles than the Control one. These trends are reproduced in the simulated data.

The left panels of Fig 5 show the difference in the risk allele load between the Case and Control populations: The Control population had more zero values, while the Case population had more “2”’s. However, this difference is not large with an average risk allele load for the Case group of 46.5 and for the Control group of 43.5, about 7% difference.

### Extending the core data

Since the core data sets are limited, and their size is too small to answer the questions we wished to explore, we did not use the core data directly, rather, we used the core data to generate sets of simulated data that were used in this study. The data were simulated such that the new Case population was derived from the core Case subset, and the new Control set was created from the core Control population. We required the simulated data sets to have a distribution as similar as possible to the distributions of risk alleles in the core data sets (in terms of the number of 0, 1, and 2’s).

**Fig 6:**
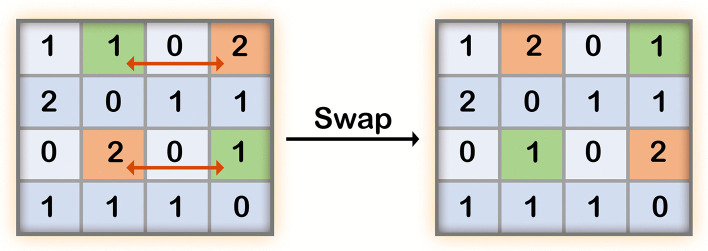
Schematic example of a single swap. Each row lists the number of alleles at each of four SNP positions in an individual (0, 1, or 2) and each column lists the number alleles for a specific SNP across four individuals. The swap is allowed as both the first and third rows have the values ‘1’ and ‘2’ in opposite positions. The result of that single swap is shown on the right: the sum of value in each row and column is maintained, while swapping the order within each row and column.

To achieve this, we devised a shuffling method, which we termed “Rectangle shuffle” that maintains the total number of 0, 1 and 2s for each risk allele over the set of individuals (column) and for each individual over the set of alleles (row), while changing the internal order of values. Thus, we swapped the positions of two values in a row simultaneously with another row that has the same values in opposite positions, such that the sum of risk allele values is preserved in all rows and columns (Fig 6).

Each such swap results in the alteration of two alleles in only two individuals. To create a new simulated set of individuals, we repeated this process (NxM)log(NxM) times on a set of size N individuals and M alleles; this number of swap operations guarantees that the resulting matrices deviate sufficiently from their origin [31].

Each simulated set of Case individuals was created from the core Case set using the “rectangle shuffle” procedure. After each shuffle epoch (i.e., (NxM)log(NxM) steps), we mapped the alleles into the disease model. Note that due to the random shuffling, an individual originating in the Case core set could become Control when mapped to a given disease model. Such individuals were rejected, and only individuals who remained with a Case phenotype were added to the new set. Remaining individuals were shuffled again. Once 80% of the population size is generated, it usually becomes increasingly difficult to produce additional new Case individuals by “rectangle shuffling”, and at that point we reverted to regular shuffling of the rows. Fig 5 shows that despite this deviation from full rectangle shuffling, we achieved populations in which the allele distribution follows that of the original population: the average risk allele load is 46.7 almost indistinguishable from the 46.5 of the core data set.

New Control sets were created in a similar way from the original Control set. Because of the parameterization of pathway disease models, we could create the entire new Control sets using rectangle shuffling, and thus the average risk allele load of 43.5 load was maintained. For different experiments, we used different population sizes by combining several new simulated datasets. We referred to the sizes as multiples of the numbers of original sets, i.e., one set (4953 individuals, 3004 Case, 1949 Control is a size of 100%), two sets (200%), three sets (300%), etc.

### Machine learning algorithms

The machine learning algorithms tested in this study were NB-Naïve Bayes, LR-Logistic Regression, DT-Decision Tree, RF-Random Forrest, NN-Neural Network). All of these algorithms were run using the WEKA 3 software (https://www.cs.waikato.ac.nz/ml/weka/) with the default parameters as set in the Weka Package. For the neural network a configuration of a single hidden layer with 25 sigmoid nodes were used. The network was fully connected, i.e., each hidden node is fully connected to all input nodes and to the output node.

### Defining disease prevalence

In the real world, different diseases have different prevalence, and we wanted our model to capture this property. Thus, we considered a disease model as “rarer” if it was more difficult to produce Case individuals for that disease than it was for another. The percentage of individuals from the original Case subset that were classified as Case compared with the number that were classified as Control was used as a measure of the rarity of the disease. For example, if before shuffling, 25% of the individuals from the core Case population were assigned as Case when the alleles were mapped to a specific disease pathway model, then we set the relative prevalence of this disease in the population at 25%. We note that unlike the real-life situation where the prevalence of rare complex trait diseases such as Crohn’s Disease is on the order of 1:1000 and there is an order of magnitude difference between rare and common complex trait diseases, in our small generic model we had to use much higher prevalence, and the prevalence of “rare” versus “frequent” diseases differs by only a factor of ~2-3.

## Results

Complex trait diseases such as Crohn’s are characterized by both a small difference in the overall risk allele load between Case and Control individuals (Fig 5), and presumably, different distribution of alleles across disease-related pathways. Hence, we use in this study simulated data that were derived from data that inherently include the risk allele load bias.

### Larger datasets improve performance

**Fig 7:**
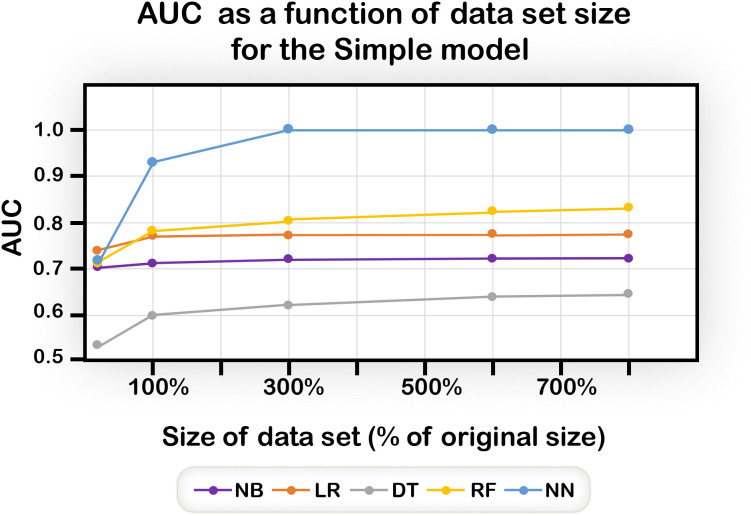
AUC of the different algorithms for the Simple model as a function of data set size. The sizes are shown on the x-axis as a percentage of the original size of 3004 Control individuals and 1949 Case individuals. All methods improve with increased data size, but the Neural Network analysis has by far the best AUC.

Performance of all the algorithms improved with increasing data size, up to a limit, as can be seen in Fig 7 for the Simple model. The NN and the RF algorithms have better performance than the others. In fact, with a population size of three times of the size of the original core data sets (namely about 9000 Control and 6000 Case), the NN reaches perfect prediction. Because of the superior NN performance (Fig 7), we used only the NN in subsequent analyses.

In order to examine the effect of the complexity of the underlying structure of the disease, we used the following three models, each testing a different type of complexity.

### The weighted model and the overlap model are more accurate than the simple one

**Fig 8:**
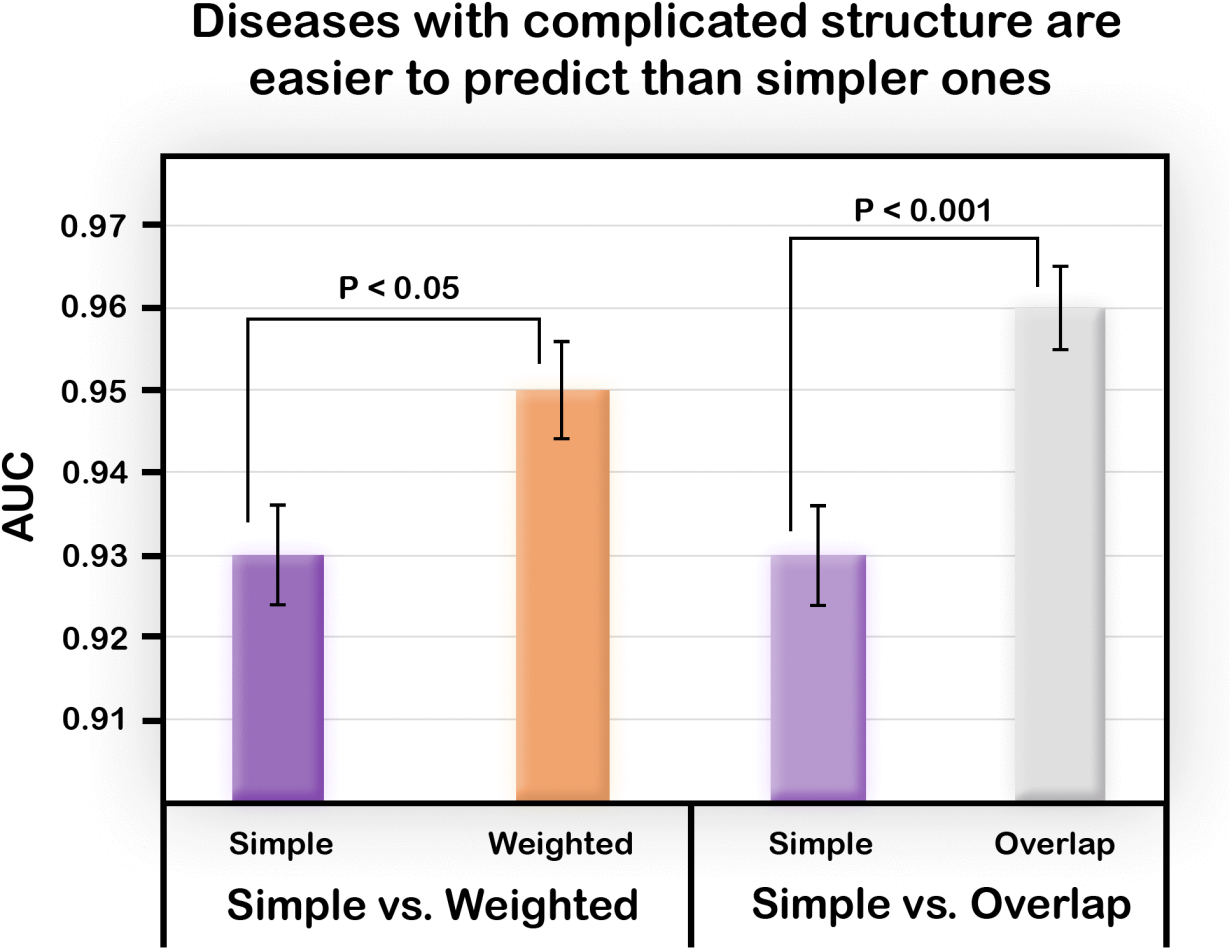
Average AUC obtained with the Weighted and Overlap models, compared with a simpler model. A more complex disease structure produces improved performance. 20 replicates were performed for each condition/model, all of size 100%. P-values of two-sample one-tailed t-test on 20 replicates for each model are shown and standard error bars are presented.

We used the Weighted model which assigns different weights to some alleles and pathways (see Methods) with the number of pathways P = 8, number of loci per pathway S = 6, the sum of risk alleles to make a pathway malfunction was set to M_g_ =20, and M_p_, the weighted sum of malfunctioning pathways required to make an individual Case, was set to 11. The number of high risk allele genes (HR) in each pathway was set to 2, with 4 low risk allele genes (LR). Additionally, there were 3 “central-pathways” (CP) and 5 “peripheral” ones (PP). The weights were as follows: HR = 5, LR = 2.5, CP = 5, PP = 2.2. We created 20 replicates for this model. The results (Fig 8, left) show that the Weighted model is better predicted than the Simple one.

Using the Overlap model defined in the Methodology section, we constructed a model with the parameters P = 8, S = 7, Mg = 5, Mp = 6, such that every path with an odd index shared two SNPs with the path in the even index that followed (paths 1 and 2, 3 and 4, etc.). This resulted in an Overlap model. Our results (Fig 8, right) showed that the performance of predicting the status of individual in the Overlap model was better than that of the Simple one.

Both the Weighted and the Overlap models add a level of complexity to the structure of the disease. Counterintuitively, this extra complexity does not make prediction less accurate, actually, the algorithm performs better.

### The subtype model is less accurately predicted than the simple model

Another form of complexity, frequently observed in clinical situations is when two diseases, each with a different genetic background, are classified phenotypically as a single disease. Examples include different molecular subtypes of breast cancer (ER + ve/ER –ve, and overexpression of HER2) [26,32,33], Parkinson’s Disease [34], and Autism [35]. Next, we tested the effect on prediction accuracy when two genetically different diseases are treated as one. To simulate this scenario, we split the 48 alleles and built a “Subtype” model consisting of two small Simple sub-models each with the parameters P = 4, S = 6, Wg =2, Wp = 5, and each using 24 alleles. For comparison purposes, we created another Simple model, which included only the first sub-model of the Subtype, i.e. using the first 24 alleles and the parameters [P = 4, S = 6, Wg = 2, Wp = 5]. We referred to this model as a “Single subtype model”.

For the “Single subtype model” (i.e., a disease model of 24 risk alleles) with population size of 100%, the NN achieved perfect AUC of 1. When the “Two subtype model” was run with a population size of 100%, its performance was poor (0.74). Nevertheless, the population size is effectively halved when we run a “Two subtype model” and thus, to attain a valid comparison, we should run the “Two subtype model” with a population of 200%. For this size, the NN achieved an AUC of 0.85. Even using a population of 400%, the performance of the “two subtype model” was only 0.89, still inferior to the AUC of 1.0 attained for the “Single subtype model”. These results suggest that when the algorithm is faced with two alternative disease models the ability to learn each model declines.

### Missing risk alleles reduce performance, while surplus ones have a lesser effect

For complex diseases, the exact list of alleles that play a role is unknown. Hence, the set chosen may lack relevant alleles, and/or include alleles that are not relevant to the disease. To examine the effect of missing alleles, we used the Simple model, and deleted various percentages of alleles that were used for determining disease status, thereby creating “missing” data sets, in which the disease status of an individual was determined by the full set of the 48 alleles but the predictive model was based on a randomly chosen subset of these alleles.

To examine the effect of surplus alleles, we again used the Simple model, this time with a variable percentage of additional alleles assigned random values (with the same distribution of 0, 1, 2 as in the original sets) that do not influence disease status.

**Fig 9:**
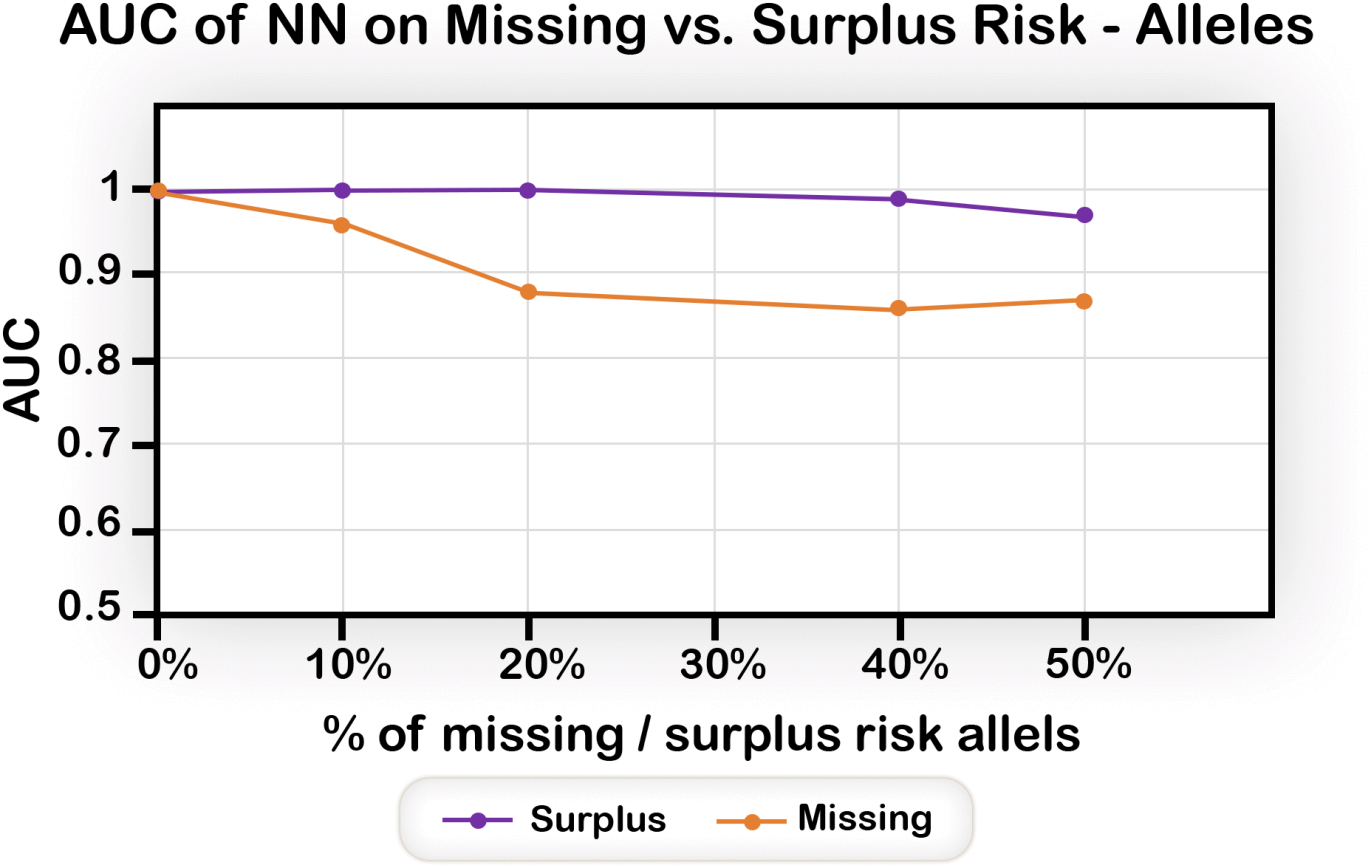
The effect of missing and surplus alleles on prediction performance. Omission of relevant alleles has a larger effect compared to the inclusion of irrelevant alleles. Average AUC, Simple model with a dataset size of 200%.

The results (Fig 9) show that the effect on performance of excluding relevant alleles is much more severe than the effect of adding non-relevant alleles. This is expected, since ML algorithms are good at selecting relevant features from a set that includes non-relevant features, but their ability to fill-in missing information is more limited.

### Mislabeled data moderately weakens performance

Complex diseases are caused by a combination of multiple genetic, environmental, and lifestyle factors. Thus, some individuals may have a genetic predisposition for a given disease, but in the absence of an external trigger (viral infection, diet, air pollution, stress) they may remain healthy. It could be claimed that the data on which the ML models were trained and tested is taken from a real population which may include many individuals who have a genetic makeup predisposing to disease but have yet to encounter the additional trigger. We next explored to what extent this situation would reduce performance in our setting.

**Fig 10:**
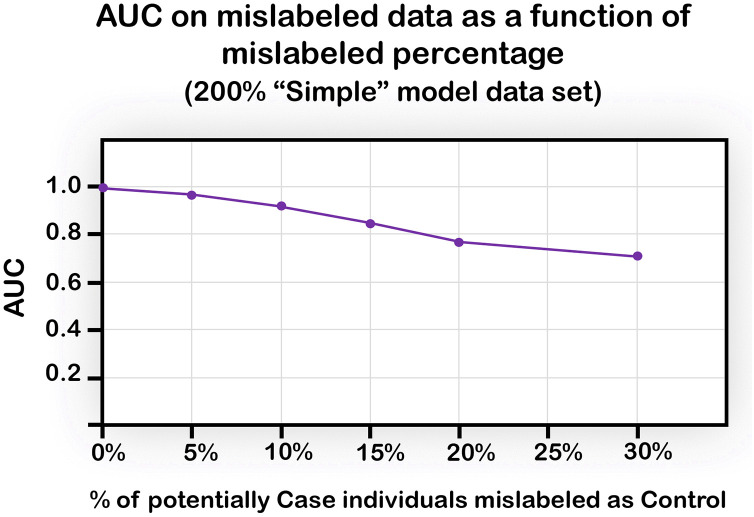
Average AUC obtained with various percentages of mislabeled individuals in the training data, using the NN algorithm. The percentage on the x-axis represents the percentage of Case individuals mislabeled as Control out of all Case individuals in the training set.

To this end, we again used the Simple model and randomly chose a varying percentage of Case individuals from the training set, and intentionally mislabeled them as Control. The results (Fig 10) show a decline that is moderate up to 10% of mislabeling, with performance still above AUC of 0.9, followed by a sharper decline in the presence of a greater percentage of mislabeled samples.

We note that we have analyzed only one type of misclassification, mistakenly considering case as control. We focused on this direction since in many complex diseases which do not manifest until later in life, the phenotypic status may be healthy for many years while the disease related genetic makeup already exists. Interestingly, a recent study by [36] showed that misclassification of the other type, labeling control as cases, has a much larger effect on the decline in classification power.

### Low prevalence disease is easier to predict than high prevalence disease

Finally, we wished to examine the effect of the “prevalence” of the disease on the performance of the algorithm. As described in the Methods, in our model, “prevalence” reflects the difficulty of producing Case individuals from the underlying risk allele frequency of the core Case dataset.

As we noted above, using this definition of prevalence and with a small generic model, the actual prevalence is much higher than in real life. We also can not directly choose the prevalence percent identified by our model. In the models we discussed so far, the prevalence ranged between 35–40%. Thus, we adjusted the parameters to attain less frequent disease prevalence, in the range of 10–29%.

**Fig 11:**
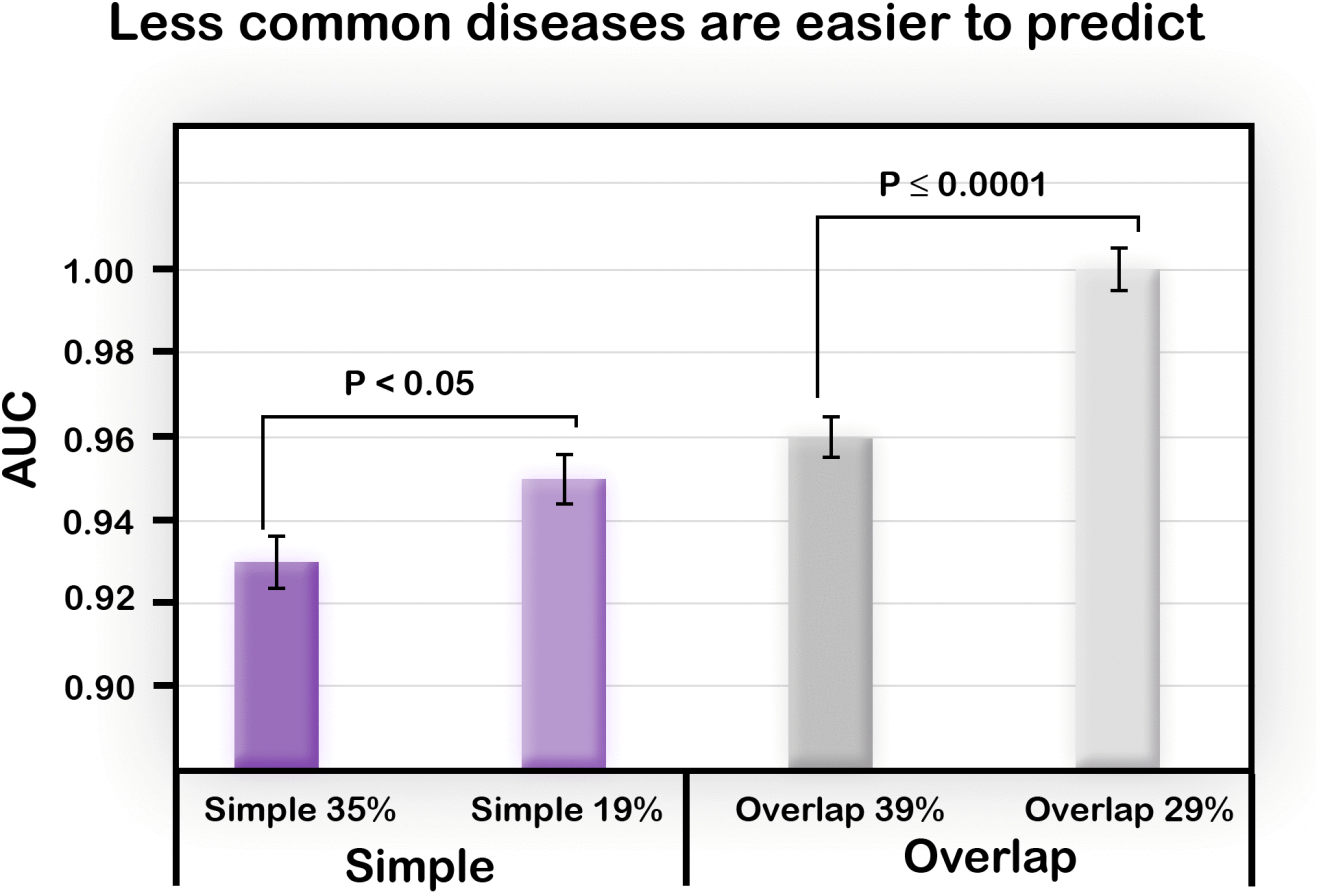
Comparison of models with different prevalence. Left: The performance on a Simple disease model with disease prevalence of 35% is compared with the performance on a Simple model in which the parameters were tuned to decrease the prevalence to 19%. Right: For the Overlap model, a disease with prevalence of 39% was compared to a model with a lower prevalence of 29%. In both cases, the performance was significantly better for the less common diseases. Population size of 100% was used, and the results are based on 20 replicates. P-values of two-sample one-tailed t-test were calculated and standard error bars are presented.

We created another Simple model, this time with parameters that resulted in a lower disease prevalence of 19%; P = 8, S = 6, Wg = 5, Wp = 5. We also tested the effect of prevalence on performance using the Overlap model described above, comparing a prevalence of 39% using the parameters P = 8, S = 7, Wg = 5, Wp = 6 with a model of 29% prevalence resulting from the parameters P = 8, S = 7, Wg = 6, Wp = 5. While within this range of parameters, the algorithm performed well in both cases (Fig 11); we can see that the less common diseases were better predicted than the more common ones.

### It is possible to gain insights into the structure of a disease from the behavior of the prediction algorithm

Assuming one can provide a well-performing model that can accurately distinguish between susceptible and non-susceptible individuals, is it possible to use the model to gain insights into the underlying genetic structure of the disease? Since in our generic models, we have defined the full genetic structure of the disease, we can try to “reverse engineer” the output of the NN models, examining the potential for using these results to infer the disease mechanisms.

The NN models used in this study were built as fully-connected networks with one hidden layer consisting of 25 neurons and with two output neurons (one for each class).

The output of the learning process is a set of edge weights. We extracted the weights that connected the input nodes (representing the 48 loci) to the hidden layer and organized them in a table ((one table for each run) where for each genomic loci we tabulated the weights that connect the input node representing the loci to each one of the 25 hidden nodes.

**Fig 12:**
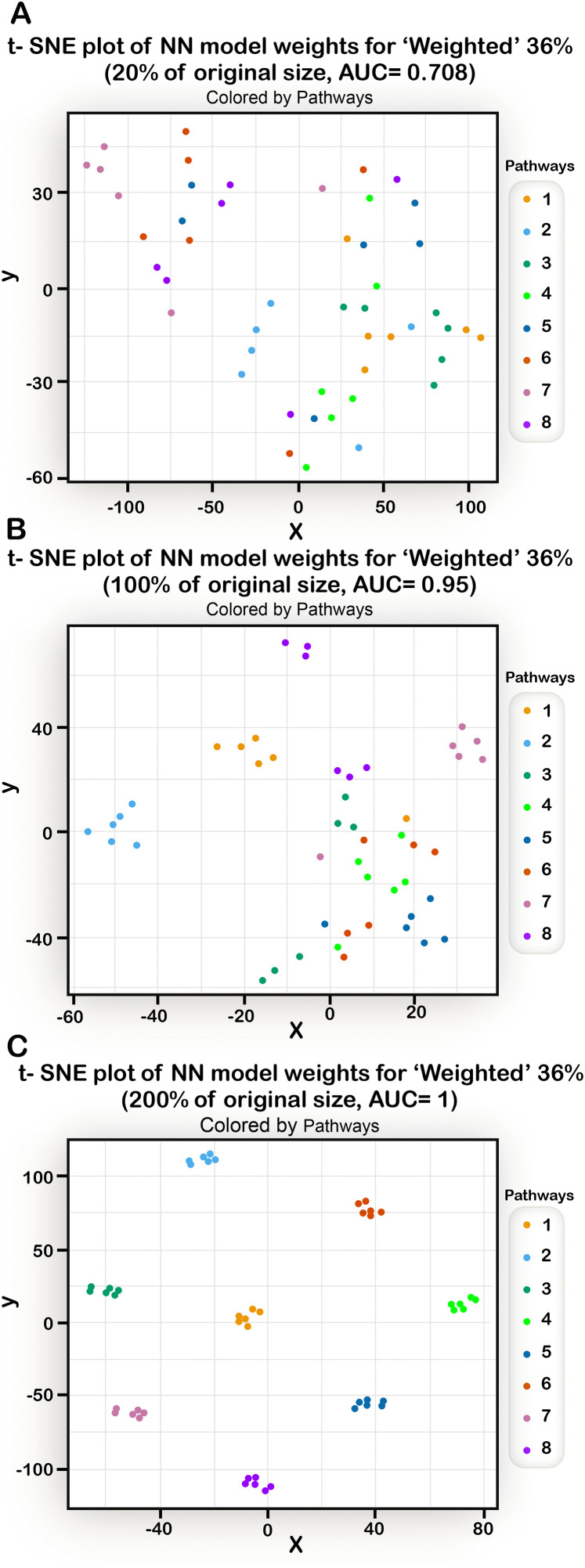
The results of running a t-SNE algorithm with NN weights as input, for different AUC value situations. Each point represents a SNP, and points are colored according to the pathway the SNP sits in. (A) t-SNE of the Weighted model (20% of the original size, with AUC of 0.708). (B) t-SNE of the Weighted model (100% of the original size, with AUC of 0.95). (C) t-SNE of the Weighted model (200% of the original size, with AUC of 1). For higher AUCs, the ability of the t-SNE algorithm to extract the structure of the underlying disease model improves significantly.

**Fig 13:**
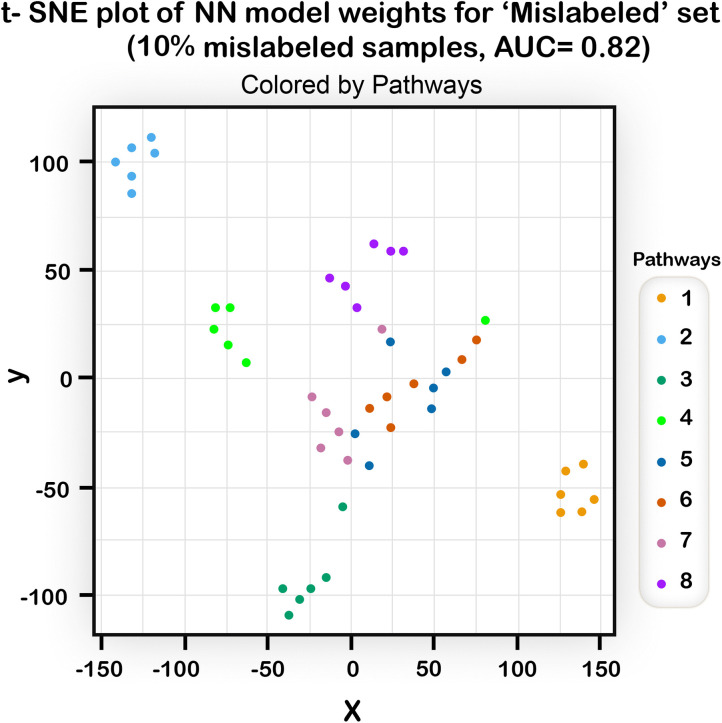
The results of running a t-SNE algorithm with NN weights of a “mislabeled” model as input (100% of the original size, 10% mislabeled samples in training set, with AUC of 0.82). Even with an AUC that is not very high, the ability of the t-SNE algorithm to extract the structure of the underlying disease model is good.

**Fig 14:**
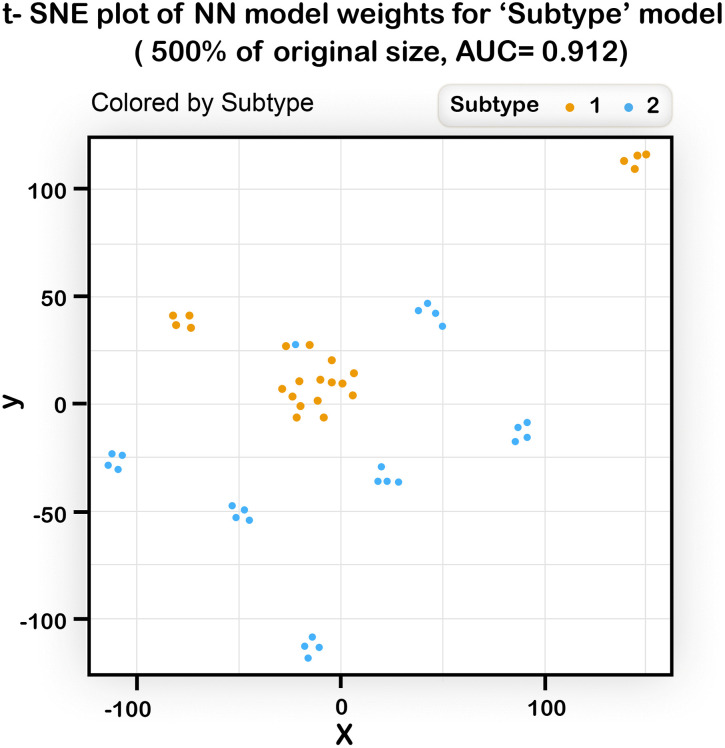
The results of running a t-SNE algorithm with NN weights of a “Subtype” model as input (500% of the original size, two subtypes model, AUC of 0.91). Predicting disease status in a subtype model was shown to be a difficult task. Still the plot shows a reasonable separation between pathways that belong to type 1 (on the upper part) and pathways that belong to subtype 2 (mostly lower part).

These tables were used as inputs to the t-SNE algorithm [37] which returns a 2- dimensional graph in which each point is a 2D representation of a row from the table, i.e.,; a representation of an allele. We then colored the SNPs according to their pathway in the disease model to see if their representation in the 2D plane segregates according to their real pathway. Several types of models were used with different AUC’s and the results of the corresponding t-SNE runs are shown in Figs 12, 13, 14.

We can see (Fig 12) that when the AUC of the NN model used as input is high enough, the graph resulting from the t-SNE shows clear clustering of the SNPs into their pathways. In another example (Fig 13) it is evident that even with an imperfect AUC, noticeable clustering is still observed. In Fig 14, the t-SNE algorithm, given a large enough data size, can identify quite well the different pathways and, in addition, it can separate between the two subtypes (as most of the pathways from subtype 2 appear in the lower part of the plot). The separation is not perfect, but given that the subtype model was difficult to predict, these results are encouraging. Taken together, the t-SNE plots demonstrate that the NN algorithm has the potential to elucidate the genetic structure of diseases.

## Discussion

Our disease models are not meant to replace the established genetic models, but rather to create a sandbox for exploring disease properties that would otherwise be difficult to study. These models are extremely simple, yet we argue that this simple framework can capture some key aspects of real diseases.

### How do the disease models used here relate to real life situations?

A dominant Mendelian disease like Achondroplasia, where a single variant in the FGFR3 gene causes a disease phenotype [38], is an example of the “Simple Model” with one pathway that includes a single gene

Digenic diseases such as Retinitis Pigmentosa type 59 [39] where deleterious variants in two genes (PRPH2 and ROM1) are required to obtain a disease phenotype, can be described in terms of the “Simple Model” with two pathways, both of which must be defective.

An example of a “weighted model” is thrombophilia where variants in the F5 and F2 genes are known to affect the Coagulation pathway, but the variant in the F5 gene [40] carries higher risk than the variant in the F2 gene [41].

An example of an “overlap model” is provided by a variant in NOD2 associated with increased risk of Crohn’s disease that may affect three different pathways involved in response to bacteria: reduced signaling from toll-like receptors, reduced NOD2 protein migration to the cell outer membrane, and reduced production of a defensin anti-bacterial peptide [42].

An example for the “subtype model” is Diffuse Large B-Cell Lymphoma (DLBCL), until recently considered a single lymphoma subtype and now is split into at least two distinct diseases: Germinal center B-cell like diffuse large B-cell lymphoma GCB) and Activated B-cell–like lymphoma (ABC) [43].

### Epistatic non-additive interactions

At first sight, this abstract model does not address important aspects of epistasis. Epistatic non-additive interactions (i.e., when the effect of variants in two genes on the phenotype is different from a simple addition of the effect of each one) may play an important role in understanding disease. For Example, a recent large-scale analysis of statistical epistasis across 70 traits using UK Biobank data found an epistatic effect on genetic variance comprising about a quarter to half that of the additive contributions, but sample size limitations prevented an accurate estimate [44]. Thus, while uncertainty remains as to the magnitude of the effect of epistasis in disease mechanisms, it does appear that many variants affect susceptibility in a non-additive manner.

Given that there are millions of SNPs and thus more than 10^12^ potential pairs of interactions, it will take much more data and/or new methodology than currently available to adequately address epistatic contributions. One such methodology is an explicit network representation following the principles outlined here.

We note that our model is inherently non-additive. Because of the threshold imposed on the ability of each pathway to maintain its function, once the threshold has been crossed, additional variants do not affect the output. Furthermore, the interplay between pathways adds another layer of non-additivity, e.g., variants that belong to two different pathways may have a very different contribution to the phenotype than if they are located in the same pathway.

In addition, we note that it is possible within the framework of the model to directly describe epistatic pairs. Epistatic variant pairs are often classified as either producing “negative epistasis” where the combined effect is smaller than the sum of the individual contribution, “positive epistasis” where the combined effect is greater than the sum of the individual contributions, and the more rare scenario [45] of “reciprocal sign epistasis” wherein the combined effect of two variants that are individually damaging is reversed, and together they become protective (or vice versa). The Supplementary material file S3 File provides a detailed description of how our current model can represent “negative epistasis” and “positive epistasis” and indicates a simple modification that is required to allow it to describe “reciprocal sign epistasis”, as well.

### Modularity

The model we present here is inherently modular, as is the case with many complex trait diseases. For example, analysis of the GWAS loci associated with Crohn’s disease risk implicates variants in genes involved in at least four principal processes: gut barrier integrity, autophagy, the innate immune response, and the adaptive immune response, with some risk alleles contributing to more than one process [46,47].

## Limitations of the model

Still, some aspects of complex trait diseases are ignored in the current model. While we consider the different components of a disease as “pathways”, they are actually “modules”, in the sense that the location of a gene within a pathway (either upstream or downstream) is not relevant. The model also does not refer to the concept of “dominance” where a heterozygous mutation in one copy of a genomic locus is sufficient to confer pathogenicity. Rather, we use the conventional scale of 0, 1 and 2 representing the risk load as a result of being wildtype, heterozygous or homozygous for a certain mutation. Including the effect of dominant mutations would require developing a model that explicitly contains two allele systems, which is beyond our current scope.

Our model does not address the issue of linkage disequilibrium (LD) in which adjacent genetic variants are correlated. A “Real world” model must address this issue to avoid double counting of the same genetic feature. Accordingly, the purpose of LD analysis is to identify a core set of loci that are independent of each other. Our model assumes that some kind of LD analysis has been performed and the small set of alleles that are incorporated are already “clean, and represent a core set of independent genetic features that are involved in disease risk.

Another major limitation is that we do not include the environmental non-genomic factors. The model is deterministic and considers only the genetic factors and not the environmental components that play significant roles in complex trait diseases [48]. Including environmental factors is difficult in controlled datasets [19] and adding such factors is not likely to change the conclusions of the study. For example, if we show that the Subtype model is more difficult to predict than a Simple model, this conclusion would stand regardless of the extent of the non-genetic components that are associated with the disease. However, the model can be extended to include non-genetic factors as well by adding randoms term to the genetic load on each pathway although further study would be needed in order to select the distribution from which to draw these random terms.

Despite the limitations mentioned earlier such as genetic loci redundancy, family structure, ethnic variation, and potential effects of population stratification we argue that our models capture key aspects of the fundamental complexity of diseases. Real-life phenotypes are partly determined by genetic interactions within and between modules, each module is characterized by a specific degree of robustness, so that a combination of genetic components is needed to produce a susceptible genotype. We have incorporated additional aspects of real diseases into different models, including varying effect sizes of the variants (Weighted model), more intricate relationships between pathways (Overlap model), different diseases with similar phenotypes (Subtype model), and the effects of misdiagnosis.

In this study, we present a “toy-model” that allows us to investigate factors that affect the ability of machine learning algorithms to predict an individual’s susceptibility to a complex trait disease based on their genotype using a simple generic model of disease. In a real world situation, it would be difficult to compare, for example, the prediction performance for a common disease to the performance of the model on a rare disease, since realistic models of such two diseases would differ by many confounding parameters and not only by disease prevalence.

Among the factors we investigated, we found, as expected, that increased dataset size enabled the NN algorithm to deliver better performance, up to a plateau, probably reflecting the point at which the algorithm has exhausted its ability to learn from this type of data. A recent study on a more realistic model of inflammatory bowel disease [18] reached a similar conclusion.

Additionally, we found that when the dataset lacks risk alleles that are relevant to the determination of disease status, the negative effect on the algorithm is much stronger than the inclusion of irrelevant risk alleles. Thus, risk alleles associated with disease at lower confidence may be included in a model with little downside. This conclusion is consistent with the current trend of using Polygenic Risk Scores (PRS) based on thousands of alleles that are, at best, weakly associated with the disease.

Counterintuitively, we saw that performance was better using the more intricate models (Weighted and Overlap) rather than the Simple ones. This observation can be attributed to the fact that these models create a subgroup of alleles that stand out from the rest (e.g., alleles with higher weights), which contribute more strongly to the disease status, and thus, contribute to performance when they are identified successfully by the algorithms. This rationale may explain why in the “Subtype” model, the performance was less good, as in this model, no alleles were singled out, and the difficulty of prediction arose from the two parallel networks.

Surprisingly we found that the rarer the disease was, the better the prediction outcome. Here too, we suggest that this effect is caused by the fact that in a rare disease, susceptible individuals carry a more unique combination of risk alleles, enabling them to be more easily separated from “Control” individuals.

Furthermore, the ability to retrieve a disease model from the neural network using the t- SNE algorithm demonstrates an advantage of using a relative small mechanistic model over Polygenic Risk Score models that are based on thousands of SNPs and are notoriously difficult to interpret [49].

In summary, we believe that the model presented here can serve as a valuable thought experiment for enhancing our understanding of the relationship between disease structure and the ability to predict disease status.

## Supporting information

S1 File Numerical Examples of the Models. (DOCX)

S2 File Glossary of Model Parameters. (DOCX)

S3 File Odd Ratio Model. (XLSX)
