# Supplementary material for: Using machine learning to predict and analyze complex trait diseases: Lessons from a simple abstract model
Source: PLoS One. 2026 Feb 23;21(2):e0342490. doi: 10.1371/journal.pone.0342490 (PMC12928469; doi:10.1371/journal.pone.0342490)
Supplement: S1 File — (DOCX) [file pone.0342490.s001.docx]

**Numerical examples:**

**
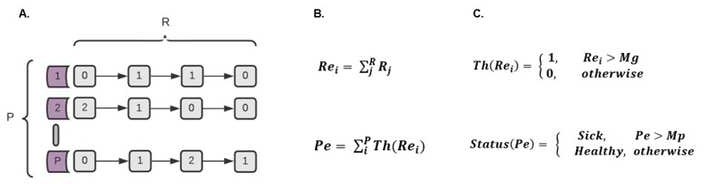
The Simple model:**

Iet’s assume that there are 3 pathways (i.e. p=3), 4 nodes in each pathway, where the risk allele status of each node is 0 (wildtype), 1 (heterozygous) or 2 (homogeneous) as depicted in the figure above. In this example Mg (the threshold for malfunction pathway) is set to be 2 (i.e. the values should be bigger than 2 for malfunction), and the threshold of the number of malfunctioning pathways for sick status (Mp) is 1.

In this example, the sum of the risk alleles along each of the pathways is 2,3 and 4, respectively. As a result, the pathways receive *Th* values of 0,1,1 respectively, and thus *Pe* is 2 which is larger than the *Mp* value of 1, and the overall *Status* of this individual is set to “*Sick*”. The individual status would be *healthy* if, for example, the first node of the second pathway was heterozygous rather than homozygous (value of 1 rather than 2), making the *Th* value of the second pathway to be 2 and thus a *Pe* of 1, not greater than the Mp value of 1.


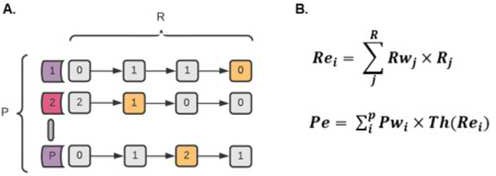
**The Weighted model:**

Assuming a similar system to the previous model (3 pathways with 4 nodes each, with the same thresholds), assume that the yellow nodes have weights of 2 while all the other nodes have a weight of 1. In this case, the values *Re* of the pathways will be 2,4,6 respectively and the corresponding *Th* values would be 0,1,1 rendering this individual to be *Sick*.


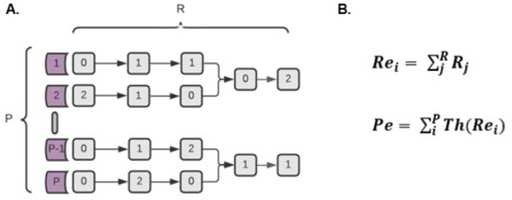
**The Overlap model:**

In this example we have 4 pathways with 5 nodes each where the last two nodes are joined between pathways 1 and 2 and between pathways 3 and 4. The *Re* values for the pathways are 4,5,5,4 respectively. If the thresholds are set to *Mg* = 4 and *Mp*=1, then the *Pe* value of 2 will result in this individual being considered *Sick*.

**
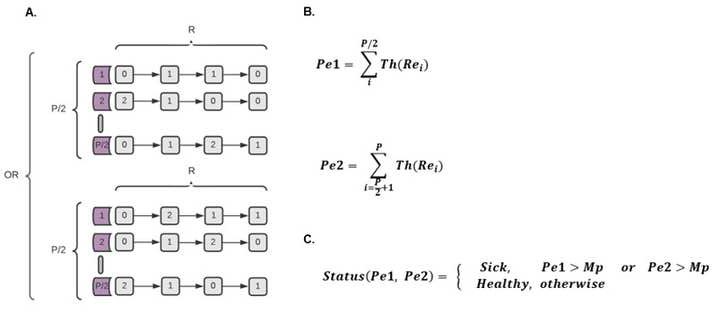
The Subtype model:**

Here we have a model with 6 pathways with 4 nodes each. The pathways are divided into two subtypes with 3 pathways in each subtype. The *Re* values for the first subtype are 2,3,4 respectively, while for the second subtype they are 4,3,4 respectively. With a threshold value of *Mg* = 3 the *Th* values for the first subtype will be 0,0,1 while the *Th* values for the second subtype will be 1,0,1. With *Mp* set to 1, this individual will be considered “Sick” because the second subtype will be above the threshold.
