# Supplementary material for: Using machine learning to predict and analyze complex trait diseases: Lessons from a simple abstract model
Source: PLoS One. 2026 Feb 23;21(2):e0342490. doi: 10.1371/journal.pone.0342490 (PMC12928469; doi:10.1371/journal.pone.0342490)
Supplement: S2 File — (DOCX) [file pone.0342490.s002.docx]

## Table: Model Parameters

| Parameter | Description |
| --- | --- |
| P | Number of disease-related pathways included in the model. |
| R | Number of genes (or loci) per pathway, |
| Rj | Numeric representation of a genotype at a locus: 0 (wildtype), 1 (heterozygous), or 2 (homozygous variant). |
| Rwj | Weight assigned to a specific risk allele, reflecting its relative contribution to disease risk. |
| Rei | Total risk allele burden within pathway i, computed as the sum or weighted sum of its allelic values. |
| Mg | Threshold above which a pathway is considered malfunctioning due to the accumulated genetic burden. |
| Mp | Threshold defining when an individual becomes a Case; disease occurs if Pe > Mp. |
| Pe | Number or weighted total of malfunctioning pathways for a given individual. |
